# Outcomes of a state-wide salt reduction initiative in adults living in Victoria, Australia

**DOI:** 10.1007/s00394-023-03210-z

**Published:** 2023-07-26

**Authors:** Kristy A. Bolton, Joseph Alvin Santos, Emalie Rosewarne, Kathy Trieu, Jenny Reimers, Caryl Nowson, Bruce Neal, Jacqui Webster, Mark Woodward, Elizabeth Dunford, Sian Armstrong, Bruce Bolam, Carley Grimes

**Affiliations:** 1 Institute for Physical Activity and Nutrition, School of Exercise and Nutrition Sciences, Deakin University, Geelong, VIC Australia; 2 The George Institute for Global Health, University of New South Wales, Sydney, NSW Australia; 3 Victorian Health Promotion Foundation (VicHealth), Melbourne, VIC Australia; 4 School of Public Health, Imperial College London, London, UK; 5 Department of Nutrition, Gillings Global School of Public Health, The University of North Carolina at Chapel Hill, Chapel Hill, USA; 6 Heart Foundation, Melbourne, VIC Australia; 7 School of Population and Global Health, University of Melbourne, Melbourne, VIC Australia

**Keywords:** Population salt reduction, Intervention, Sodium excretion, Salt consumption, Diet, Urinary sodium, Adults, Purchasing origin

## Abstract

**Purpose:**

To assess any effects of a state-wide sodium reduction intervention on sodium intake, sources of dietary sodium and discretionary salt use at a population level.

**Methods:**

Data (24-h urinary sodium excretion, self-report survey, a 24-h dietary recall) were collected cross-sectionally at baseline (2016/2017) and follow-up (2020) from adults in Victoria, Australia. Intervention activities included consumer awareness advertising campaign, public debate generation via mass media, strengthening existing policy initiatives and supporting food innovation with industry.

**Results:**

There were 339 participants at baseline and 211 at follow-up, with 144 and 90 of participants completing a 24-h dietary recall, respectively. There was no difference in adjusted 24-h urinary sodium excretion between baseline and follow-up (134 vs 131 mmol/24 h; *p* = 0.260). There were no differences in the percentage of participants adding salt during cooking (63% vs 68%; *p* = 0.244), adding salt at the table (34% vs 37%; *p* = 0.400) or regularly taking action to control salt/sodium intake (22% vs 21%; *p* = 0.793). There were large differences in the quantity of dietary sodium sourced from retail stores (57% vs 77%, *p* < 0.001), and less sodium was sourced from foods at fresh food markets (13% vs 2%; *p* ≤ 0.001) at follow-up. No large differences were apparent for foods with different levels of processing or for food groups.

**Conclusion:**

There was no clear population-level effect of the 4-year multi-component Victorian Salt Reduction Intervention on sodium intake with Victorian adults continuing to consume sodium above recommended levels. The findings indicate that more intensive and sustained efforts aiming at the retail and food industry with national level support are likely to be required to achieve a measurable improvement in sodium intake at a state level.

**Supplementary Information:**

The online version contains supplementary material available at 10.1007/s00394-023-03210-z.

## Background

The adverse impact of overconsumption of sodium (salt) on health and the economy is well known [1]. High salt intake raises blood pressure, which is the major cause of cardiovascular disease (CVD) [1]. In Australia, 4 million Australians live with CVD [2, 3]; and in 2019 CVD was the underlying cause of 26% of all deaths [3, 4]. The World Health Organization (WHO) suggests that adults should limit salt consumption to less than 5 g per day (g/d) [5], yet a recent Australian systematic review estimates salt intake to be, on average, 9.6 g/d [6]. As a member state of the WHO, Australia has committed to achieving a 30% reduction in population salt intake by 2025 [7, 8]. Population approaches to salt reduction have been promising in the UK [9], Finland [10] and Canada [11]; and modelling indicates them to be cost effective or cost saving [12] and economically feasible in reducing the burden of non-communicable disease [13]. However, despite this, efforts to reduce salt in Australia, to date, have been limited [14].

Approximately a quarter of Australia’s population live in the state of Victoria [15]. To address the overconsumption of salt in Victoria, the Victorian Salt Reduction Partnership was formed in 2015 [16]. This was a partnership between key stakeholders from health-related non-government and government organisations, and the academic sector (Heart Foundation, VicHealth, Victorian Department of Health, The George Institute for Global Health and Deakin University’s Institute for Physical Activity and Nutrition). The Victorian Salt Reduction Partnership set an aspirational goal of reducing salt intake by 1 g/d in adults over 4 years (by 2020) [17]. There were four main intervention components: (1) raising consumer awareness to improve attitudes and change behaviours related to salt intake (e.g. consumer awareness advertising campaigns), (2) generating public debate (through mass media advocacy), (3) strengthening existing policy initiatives (e.g. state government institutional nutrition policies), and (4) supporting food innovation (e.g. engaging food manufacturers, developing case studies of reformulation progress and successful reformulation guidance for food manufacturers) [18, 19]. Evaluation of the initiative was comprehensive over the duration of the project 2015–2019 (extended to 2020) [17]. This included examination of the impact in primary-school aged children [20]; stakeholder interviews [21–23]; process and economic evaluation; each of which will be reported separately.

The aim of the current study was to assess any effects of a state-wide sodium reduction intervention on sodium intake, sources of dietary sodium (food groups, NOVA level of processing, origin of purchase) and discretionary salt use at a population level; and ultimately determine if the Victorian Salt Reduction Partnership’s aspirational target to reduce salt intake by an average of 1 g/d in Victorian adults by 2020 was achieved.

This evaluation is important as findings can inform the design of future salt reduction initiatives nationally and internationally.

## Methods

Data were collected cross-sectionally, at baseline in 2016/2017 [24] and follow-up in 2019/2020. The methodology for data collection at both time points has been previously described in detail [24] and is briefly summarised below.

### Sample size

A sample size of 400 at each time point was required to have at least 90% power to detect a ≥ 1 g/d difference in salt intake over time, based on a mean salt intake of 9 g/d in adults [25]. The sample size calculated at each time point was not achieved and a post hoc power calculation was conducted using the actual data collected and the *simr* package in R [26]. This re-estimation revealed that the final sample sizes at baseline and follow-up, reported in the results section, were sufficiently powered to detect the 1 g/d change (based upon 100 simulations, power was approximately 100%).

### Participant recruitment

The sample for each time point of this pre-/post-study design aimed to be reflective of the state-wide adult population stratified by age and sex with recruitment strategies designed to achieve this [27]. Baseline participants were recruited from a previous study conducted in 2014 (if they had consented to future studies) [28], via random selection from the Victorian electoral roll and during the university orientation week held at both an urban and regional Deakin University campus (convenience sampling to recruit younger adults) [24]. Follow-up participants were recruited from the 2016/2017 study (if they had consented to future studies), a new random selection from the Victorian electoral roll and during 2020 university orientation week. Individuals were eligible if they were ≥ 18 years but were excluded if they were > 65 years old, did not live close to the Australian accredited commercial pathology service provider used for urinalysis, or were undergoing chemotherapy treatment.

All potential participants received a letter of invitation, consent form and plain language statement. Participants provided written informed consent. A subsample of consenting participants (also stratified by age and sex) were invited to conduct a 24-h dietary recall via telephone. Upon completion of the 24-h urine collection and survey, a $20 supermarket voucher was mailed to the participant in appreciation for their time.

The original plan was to replicate the baseline mail out process and invite the same number of participants; however, the state of Victoria was put into Covid-19 lockdown (stay at home orders) on 30 March 2020 which significantly reduced the mail out period. Data collection for follow-up had begun late 2019 and continued intensively until 29 March 2020 when the Covid-19 pandemic began to significantly impact the Victorian population, with orders to stay at home except for four reasons (i.e. for food and supplies, medical care, exercise and essential work/education that cannot be conducted from home). Recruitment was impacted by participant’s ability to leave home to return urine collections to pathology providers. Follow-up data collection was completed in August 2020.

### Survey data collection

A hard copy self-reported survey collecting demographic data (age, sex, postcode), height and weight, medications, supplements and discretionary salt use was mailed to consenting participants along with urine collection consumables. The survey was returned via reply paid envelope. Three questions assessed discretionary salt use regarding whether the participant usually added salt at the table, usually added salt to food whilst cooking, or whether they were doing anything regularly to control their salt or sodium intake.

### 24-h urine collection

A urine collection package was sent to consenting participants which contained urine collection consumables for 24-h and spot collection, instructions and a timesheet (date, time, and quantity of any urine not collected). Participants were asked to collect urine over 24-h on a day that suited them with the collection commencing after the first morning void. Participants lodged their collection at their local Dorevitch Pathology centre for analysis. Spot (50 ml) collections were included within the 24-h collection period, and the volume of urine and sodium excretion were summed. Sodium content was determined using an ion selective electrode, and creatinine was determined using Jaffe alkaline picrate, kinetic with black rate correction methodology in a Siemens ADVIA 2400 autoanalyser.

### 24-h dietary recall data

A 24-h dietary recall was administered over the telephone within 2 weeks of urine collection, dependent on participant availability. At both time points, it was aimed to collect data from a subsample of 200 participants reflective of the age and sex of the Victorian population. A five-pass method was utilised and included: (1) quick list, (2) forgotten foods, (3) time and occasion, (4) detail cycle and (5) final probe [29], with participants estimating portion sizes during the recall by using the Australian Health Survey food model booklet [30] which had been previously mailed to them. The question “where did you get this/most of the ingredient for this (food name)?” determined the source [31], with response categories tailored to the Australian context. Response categories included store (e.g. grocery, supermarket, convenience, specialty); fresh food market (e.g. butcher, local/farmers/fruit/vegetable market, green grocer); quick service restaurant or takeout/delivery (e.g. fast food chains or takeaway); full service restaurant (e.g. sit-down restaurant, café); bar or tavern; vending machine; sport, recreation or entertainment facility (e.g. sporting clubs, movies, music venue); grown or caught; from someone else/gift; water from tap; do not know; other, please specify. Data were entered into FoodWorks version 8 (Xyris). If mixed dishes were reported, they were either mapped to the best match mixed dish equivalent food code available in the Australian nutrition composition database (AUSNUT 2011–2013) [32], or the disaggregated ingredients in the recipe and subsequent portion consumed was entered into Foodworks [24]. Further details on recipe management have been previously described [24]. Discretionary salt added by the participants was not entered into Foodworks, due to inconsistencies in quantifying amounts.

### Data management and statistical analysis

All data were analysed in StataSE version 15.0 (StataCorp LLC). Given that the aim was to examine sodium/salt consumption in a sample that was reflective of the age/sex distribution of the Victorian population; the sample was sex- and age-weighted using census data for Victoria (2016) [27]. Two weightings were used, depending on the subsample for analysis—one for urine completers, and one for dietary recall completers. All analyses were conducted using the *pweight* command in Stata software, and a *p* value of < 0.05 was considered statistically significant.

#### Demographic and survey data

Body mass index (BMI) was calculated using self-reported data [weight (kg)/height (m^2^)] and categorised into anthropometric status categories as per WHO definitions [33]. A participant’s level of social disadvantage was determined using the Socioeconomic Index for Areas Index of Relative Socioeconomic Disadvantage (SEIFA), at postcode level [34]. Descriptive statistics [mean, standard deviation, 95% CI, *n*, percentage (%)] were calculated.

Responses for discretionary salt use questions were dichotomised into yes (always/often/sometimes) and no (never/rarely). Do not know responses were excluded from analysis. Logistic regression models adjusted for age, sex and socioeconomic disadvantage were used to determine the change in the percentage of participants who reported discretionary salt use behaviours from baseline to follow-up. Post-estimation was used to derived adjusted percentages.

#### Urine collection data

Urine collection times were standardised to a 24-h period. Urine completeness was assessed using creatinine excretion [35]. Under and over collection of urine was assessed using previously published criteria [24, 25, 28, 36] and included: creatinine excretion (females < 4 mmol/24-h; males < 6 mmol/24-h); extreme outliers creatinine excretion (> 3 SD from sex-specific mean); urine volume of < 500 ml and more than one void reported missing (> 300 ml). The molecular weights of Na (23 g/mol) and sodium chloride (58.5 g/mol) were used to convert mmol to mg. The percentage (%) of adults exceeding the WHO guideline of < 2000 mg/d Na (salt equivalent 5 g/d) per day was calculated. The sample’s median sodium intake was also examined in conjunction with the National Health and Medical Research Council’s Suggested Dietary Target (SDT) for sodium [86 mmol/d (2000 mg/d Na)] [37].

Mixed effects regression models were used to determine the difference in sodium (salt equivalent) intake assessed by 24-h urinary excretion between baseline and follow-up. Prior to conducting the regression analysis, diagnostics to check model assumptions were conducted to examine normality of data—including visual representation of normality of data using histograms. The model was adjusted for fixed effects that have previously been associated with salt intake: age, sex, socioeconomic disadvantage, BMI and day of urine collection (week day vs weekend). Random effects were included in the model to account for those (*n* = 218) who participated at both baseline and follow-up. The analysis was conducted initially without adjustments (model 1 unadjusted); adjusting for age, sex, BMI, SEIFA (model 2 adjusted) and adjusting for age, sex, BMI, SEIFA and weekend/weekday collection (model 3 adjusted).

#### Dietary recall data

Each reported food item was matched to a code in the AUSNUT 2011–2013 food composition database [32], and sodium and energy intake was calculated. Body mass ratio (BMR) was estimated using the Schofield equation [38] and the Goldberg method (i.e. ratio of energy intake/estimated BMR) used to identify potential under-reporters for energy intake with appropriate cut-off values for the sample size [39]. Prior to exclusion from analysis, individual dietary records of under-reporters were assessed by three researchers (KAB, CG, CN), two of whom are dietitians to determine whether or not to exclude the participant. Using their professional judgment with additional information on the types and amounts of food eaten, and the participant’s response to “Was the amount of food that you ate yesterday much more than usual, usual, or much less than usual?” a final decision for exclusion based on under-reporting was made. The AUSNUT 2011–2013 database contains 2 digit and 3 digit numeric classification codes to categorise food items into major and sub-major food groups, respectively [32]. The NOVA classification system, which categorises foods into four categories based upon their level of processing (minimally processed, processed, processed culinary ingredient, ultra-processed) [40], was applied to food items in the AUSNUT 2011–2013 food composition database as previously described [41]. In addition foods consumed were categorised as core or discretionary as defined by the Australian Guide to Healthy Eating [42, 43]. The mean ratio method was used to examine the contribution of sodium from different food groups (i.e. major, sub-major, NOVA, core and discretionary) and origin of purchase of food [44]. Differences in nutrient intake and sources of sodium across time points were assessed with linear regression models adjusted for age, sex, BMI, socioeconomic disadvantage, energy intake (kj/day) and day of diet recall (week day vs weekend). The analysis was conducted initially without adjustments (model 1 unadjusted); adjusting for age, sex, BMI, SEIFA (model 2 adjusted) and adjusting for age, sex, BMI, SEIFA and weekend/weekday collection and energy intake (model 3 adjusted).

### Ethical approval

The Faculty of Health Human Ethics Advisory Group at Deakin University approved this work (HEAG-H 71_2016).

## Results

### Response rates and final participant sample

At baseline, as previously described [24], 6169 were invited and 462 participated resulting in a 7.5% response rate. The response rate at follow-up was similar, with 3539 invited, 300 participated resulting in an 8.5% response rate (Supplementary Table 1). Supplementary Fig. 1 contains an in-depth summary of recruitment, consent and participation. At baseline, of the 365 consenting participants, 26 were excluded from analysis [incomplete data collected (*n* = 10), urine sample lost (*n* = 9) and met urine exclusion criteria (*n* = 7)] leaving 339 valid urine collections. At follow-up, of the 223 consenting participants, 12 were excluded from analysis [participant out of age range (*n* = 1), incomplete data collection (*n* = 5), met urine exclusion criteria (*n* = 6)], leaving 211 valid urine collections. Of the 155 participants that completed a dietary recall at baseline, four were identified as under-reporters and seven did not report body weight therefore under-reporting status could not be determined and were excluded from analysis, leaving 144 valid dietary recalls. At follow-up, of the 97 participants that completed a dietary recall, 7 were identified as under-reporters and excluded from analysis leaving 90 valid dietary recalls.

### Impact of Covid-19 on follow-up data collection

Sensitivity analysis was conducted on follow-up data collected pre-Covid-19 pandemic prior to 30 March 2020 (65% of urine collections, 57% of 24-h dietary recalls, Supplementary Table 2, Supplementary Table 4), and there was no statistically significant difference between pre- and post-Covid-19 data collected. All analyses presented below include the full sample, including data collected pre- and during Covid-19 lockdown restrictions.

### Demographic characteristics of the sample

Table 1 presents the demographic characteristics of participants at baseline and follow-up. There were no statistically significant differences in demographic variables between participants at baseline and follow-up (including when stratifying by diet recall status) except for age. Overall, and by diet recall status, participants at follow-up were younger (*p* < 0.05), by an average of approximately 2 years in the overall sample and 7 years in the diet recall sample. Over two-thirds of participants collected urine on a week day. A higher percentage of participants at follow-up completed a dietary recall on a weekday (72.7% baseline vs 88.9% follow-up, *p* < 0.003).

**Table 1:** Demographic characteristics of a sample of Victorian adults aged 18–65 years who completed data collection (weighted)

	Urine collection completers			24-h diet recall completers			Victorian population (%) 49% males, 51% females^a^
	Baseline *n* = 339	Follow-up *n* = 211	*p* value	Baseline *n* = 144	Follow-up *n* = 90	*p* value	
	Percentage or mean (SD)	Percentage or mean (SD)		Percentage or mean (SD)	Percentage or mean (SD)		
Age (years)*	47.6 (13.4)	45.6 (13.9)	0.034	45.2 (13.1)	38.1 (13.1)	0.004	37 (median)^a^
Age group (years)			0.021			0.004	
18–24	13.0	12.4		7.7	22.3		13.3^b^
25–34	15.4	28.5		15.3	26.6		15.7
35–44	22.8	17.2		23.5	17.1		13.4
45–54	23.7	20.0		27.9	17.1		12.8
55–65	25.1	21.9		25.6	16.9		11.2
Sex (female)	53.6	49.7	0.414	56.4	46.2	0.159	51.0
BMI*^c^	25.3 (4.2)	25.3 (4.4)	0.947	24.7 (4.2)	24.3 (3.7)	0.499	
BMI category^d^			0.781			0.286	
Underweight	3.1	2.2		4.3	5.6		2.3^c^
Healthy weight	48.6	54.4		46.7	58.9		37.7
Overweight	37.8	30.8		39.7	27.0		30.6
Obese	10.5	12.6		9.3	8.5		19.1
Socioeconomic disadvantage (quintiles)			0.669			0.409	
1st quintile (greatest disadvantage)	6.7	6.2		7.3	3.3		
2nd quintile	6.3	9.4		5.3	10.6		
3rd quintile	10.4	11.4		14.2	10.7		
4th quintile	32.6	28.2		22.9	25.1		
5th quintile (least disadvantage)	44.0	44.8		50.3	50.3		
Day of data collection (weekday)	66.7	68.8	0.624	72.7	88.9	0.003	

*Mean (SD). Difference tested by independent *t* test or Chi-square test

^a^Data taken from Australian census[27]

^b^Data taken from Australian Bureau of Statistics 2016 [62]. Note this statistic includes 15–24 year olds living in Victoria

^c^Data from Victorian Population Health Survey 2016 [63]. Note the proportion does not add to 100% due to responses such as ‘don't know’ or ‘refused’

^d^Weight information missing for 20 participants

### Analysis of urinary excretion data over time

In both unadjusted and adjusted models, there was no statistically significant difference in salt intake between baseline and follow-up (Table 2). At both time points, mean salt intake exceeded WHO’s recommended limit of 5 g salt/d—at baseline, the average salt intake for participants was 7.8 g/d, and at follow-up, 7.7 g/d. If including 10% adjustment for non-urinary losses, this would equate to 8.6 g/d and 8.5 g/d, respectively. Additionally, the median sodium excretion exceeded the NHMRC SDT of 86 mmol/d with 120.9 mmol/d (baseline) and 130.5 mmol/d (follow-up).

**Table 2:** Urinary electrolyte excretion and dietary intake in a sample of Victorian adults aged 18–65 years (weighted)

Urinary excretion data	Total mean (95% CI)	Baseline mean (95% CI)	Follow-up mean (95% CI)	Regression analysis to test for differences baseline and follow-up		
				Unadjusted Model 1 *N* = 550	Adjusted Model 2 *N* = 529	Adjusted Model 3 *N* = 529
	*n* = 550	*n* = 339	*n* = 211	Coef (95% CI) *p* value	Coef (95% CI) *p* value	Coef (95% CI) *p* value
Sodium (mmol/24-h)	133 (128–138)	134 (127–142)	131 (124–139)	− 2.68 (− 11.29–5.93) 0.541	− 4.95 (− 13.74–3.83) 0.269	− 5.05 (− 13.82–3.73) 0.260
Salt (g/day)	7.8 (7.5–8.1)	7.8 (7.4–8.3)	7.7 (7.2–8.1)	− 0.16 (− 0.66–0.34) 0.541	− 0.29 (− 0.80–0.22) 0.269	− 0.29 (− 0.81–0.22) 0.260
Sodium (mmol/24 h) Median (IQR)	126 (86–171)	121 (87–170)	131 (86–171)			

Diet recall	Total mean (95% CI)	Baseline mean (95% CI)	Follow-up mean (95% CI)	Regression analysis to test for differences baseline and follow-up		
				Unadjusted Model 1 *N* = 550	Adjusted Model 2 *N* = 529	Adjusted Model 3 *N* = 529
	*n* = 234	*n* = 144	*n* = 90	*n* = 234	*n* = 229	*n* = 229
Sodium DR (mmol/24-h)	119 (111–127)	115 (106–124)	125 (111–139)	5.89 (− 6.51–18.28) 0.352	3.51 (− 8.88–15.90) 0.579	3.29* (− 8.20–14.78) 0.575
Salt DR (g/day)	7.0 (6.5–7.4)	6.7 (6.2–7.2)	7.3 (6.5–8.1)	0.34 (− 0.38–1.07) 0.354	0.20 (− 0.52–0.93) 0.581	− 0.19* (− 0.48–0.86) 0.577
Energy DR (kJ/day)	10,215 (9748–10,681)	9932 (9480–10,384)	10,597 (9707–11,486)	340.86 (− 428.15–1109.86) 0.385	187.52 (− 547.56–922.60) 0.617	262.14 (− 468.76–933.04) 0.482

Mixed regression analysis: model 1 unadjusted; model 2 adjusted for age, sex, BMI, SEIFA; model 3 adjusted for age, sex, BMI, SEIFA and weekend/weekday collection

DR: diet recall by 24-h dietary recall

*Note for diet recall sample, model 3 is also adjusted for energy intake (kj/day)

### Analysis of 24-h dietary recall data over time

In both unadjusted and adjusted models, there were no statistically significant differences in salt between baseline and follow-up (Table 2). Mean salt intake from food and beverage sources was 6.7 g/d at baseline and 7.3 g/d at follow-up.

### Analysis of 24-h dietary recall data over time (continued)

At follow-up, mixed dishes with cereal as the major ingredient; coffee and coffee substitutes and sweet biscuits contributed significantly more daily sodium compared to baseline (*p* < 0.05) (Table 3). Conversely, processed meat, English-style muffins, flat breads, savoury and sweet breads, and eggs contributed significantly less daily sodium at follow-up compared to baseline (*p* < 0.05). The food sources of sodium by major groups are presented in Supplementary Table 3.

**Table 3:** Contribution to daily sodium intake by sub-major food groups as reported by 24-h dietary recall

Sub-major food group name	Baseline (*n* = 143)				Follow-up (*n* = 90)				*p* value*
	Rank	% of daily intake	95% CI		Rank	% of daily intake	95% CI		
Regular breads/bread rolls	1	10	8.2	12.5	2	8	5.8	10.8	0.262
Mixed dishes (cereal major ingredient)	2	8	5.1	11.3	1	13	8.5	17.6	0.035
Processed meat	3	6	3.4	7.5	7	3	0.8	4.9	0.035
English-style muffins, flat breads, and savoury and sweet breads	4	5	3.1	7.5	11	2	0.9	3.9	0.049
Gravies and savoury sauces	5	5	2.4	7.2	3	5	2.4	7.9	0.478
Cheese	6	4	3.1	5.5	4	4	2.1	5.8	0.862
Cakes, muffins, scones, cake-type desserts	7	4	2.5	5.6	5	4	1.9	5.5	0.644
Mixed dishes (beef, sheep, pork or mammalian game major component)	8	3	1.7	4.7	9	3	0.8	4.9	0.712
Mixed dishes (poultry/feathered game is major component)	9	3	1.2	4.6	8	3	1.5	4.5	0.781
Dairy milk (cow, sheep and goat)	10	3	2.2	3.4	12	2	1.0	2.6	0.495
Pastries	11	3	1.1	4.2	6	4	1.0	6.9	0.764
Waters, municipal and bottled, unflavoured	12	2	1.9	2.7	10	3	1.9	3.1	0.189
Poultry and feathered game	13	2	0.9	3.3	28	0	0.1	0.7	0.059
Fin fish (excluding commercially sterile)	14	2	0.9	3.3	23	1	0.2	1.6	0.094
Breakfast cereals, ready to eat	15	2	0.9	2.5	24	1	0.3	1.3	0.150
Sausages, frankfurts and saveloys	16	2	0.5	2.9	25	1	− 0.2	1.7	0.270
Coffee and coffee substitutes	17	1	0.9	1.9	13	2	1.5	3.1	0.008
Packed (commercially sterile) fish and seafood	18	1	0.5	2.4	14	2	0.4	3.0	0.687
Pickles, chutneys and relishes	19	1	0.5	2.1	17	1	0.2	2.2	0.717
Sweet biscuits	20	1	0.5	1.9	15	2	0.2	3.0	0.028
Savoury biscuits	21	1	0.6	1.6	18	1	0.2	1.9	0.298
Yoghurt	22	1	0.5	1.5	26	1	0.2	1.2	0.974
Eggs	23	1	0.5	1.3	29	0	0.0	0.8	0.018
Beef, sheep and pork, unprocessed	24	1	0.5	1.3	27	0	0.1	0.8	0.050
Salad dressings	25	1	0.0	1.6	19	1	0.2	2.7	0.160
Batter-based products	26	1	0.0	1.4	22	1	0.0	2.0	0.742
Pasta and pasta products (without sauce)	27	1	− 0.1	1.5	20	1	0.3	2.4	0.260
Dips	28	1	0.3	1.1	16	2	0.0	3.6	0.091
Nuts and nut products	29	1	0.2	1.0	21	1	0.3	2.0	0.139

Data are only included if it contributes to ≥ 1% of intake at a baseline

*Data displayed were calculated as mean ratio and utilised in a mixed regression analysis adjusting for age, sex, BMI, SEIFA, energy intake (kj/day) and weekend/weekday collection

**Fig. 1:**
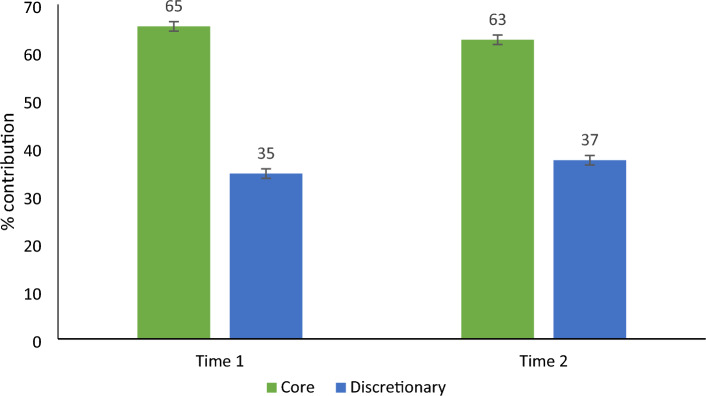
Contribution of sodium by core and discretionary categorisation as reported by 24-h dietary recall. Data are mean ± SE. Baseline *n* = 143, follow-up *n* = 90. Note no difference in energy intake by core and discretionary classification (data not shown)

**Fig. 2:**
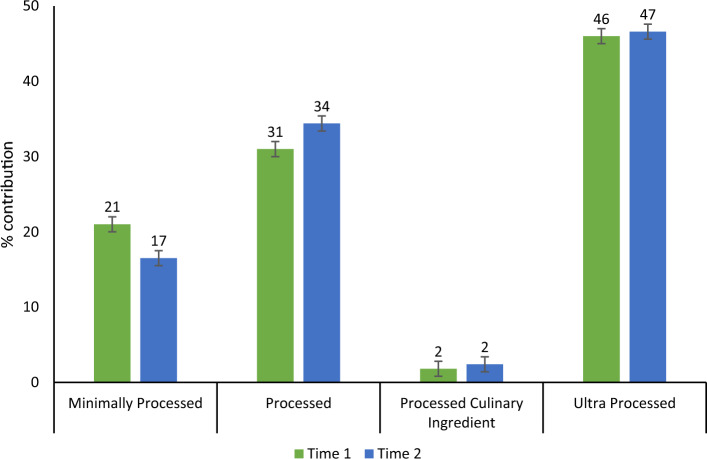
Contribution of sodium by level of processing as reported by 24-h dietary recall. Foods were categorised according to the NOVA food group classification [40, 41]. Data are mean ± SE. Note no difference in energy intake by NOVA classification (data not shown)

Examination of food categorised as core or discretionary (Fig. 1) revealed that just over two-thirds of all sodium came from core foods at both time points with no significant change over time. Further examinations of foods by level of processing (Fig. 2) revealed almost identical percentage of sodium contributing to daily intake by the four processing categories as reported by 24-h dietary recall at baseline and follow-up. Ultra-processed foods contributed almost 50% of daily sodium.

The top three origins of purchase contributing to sodium intake (baseline vs follow-up) were grocery/supermarket stores (57% vs 77%), quick service/take-out/delivery (9% vs 12%), full service restaurant (9% vs 5%) (Table 4). Whilst grocery/supermarket stores contributed the highest percentage of daily sodium intake at both time points, it contributed over a third more at follow-up (57% at baseline vs 77% at follow-up, *p* < 0.001). Conversely, at follow-up, fresh food market contributed 11% less at follow-up (13% baseline vs 2% follow-up *p* < 0.001).

**Table 4:** Origin of purchase of food and beverages consumed contributing to sodium intake reported by 24-h dietary recall

Origin of purchase	Baseline (*n* = 143)			Follow-up (*n* = 90)			*p* value*
	% of daily intake	95% CI		% of daily intake	95% CI		
Store-grocery/supermarket	57	50.9	62.7	77	70.6	84.2	< 0.001
Quick service/take-out/delivery	9	4.3	13.0	12	5.9	17.0	0.433
Full service restaurant	9	4.7	12.3	5	1.7	8.5	0.235
Water from tap	1	1.4	2.0	2	1.6	2.7	0.036
Fresh food market	13	8.7	16.2	2	0.4	3.4	< 0.001
From someone else/gift	3	1.3	4.6	1	0.1	1.2	0.021
Bar or tavern	1	0.0	1.2	1	− 0.1	1.7	0.117
Grown or caught	3	1.3	4.9	0	− 0.1	0.4	0.004

Baseline totals 95% rather than 100% due to missing origins

*Data displayed were calculated using mean ratio method and utilised in a mixed regression analysis adjusting for age, sex, BMI, SEIFA, energy (kj/day) and weekend/weekday collection. Note energy intake followed the same pattern as sodium at baseline and follow-up (data not shown)

### Analysis of discretionary salt use behaviours over time

There were no statistically significant differences in discretionary salt use over time in adjusted analyses (Table 5). There was no change in the percentage of participants who reported taking any action to control their salt/sodium intake over time.

**Table 5:** Discretionary salt use behaviours over time (weighted)

Discretionary salt use	Baseline *n* = 339	Follow-up *n* = 211	*p* value
	% 95% CI	% 95% CI	
Add salt during cooking (yes)	63 57–68	68 61–74	0.244
Place a shaker on the table at meal times (yes)	34 28–39	37 30–44	0.400
Doing anything on a regular basis to control salt or sodium (yes)	22 18–27	21 15–27	0.793

Logistic regression analysis: adjusted for age, sex, BMI, SEIFA, post hoc estimations used to derive adjusted percentage

## Discussion

This state-wide initiative had no detectable impact on overall sodium intake at a population level, nor did it have any substantive effect on the discretionary salt use or the main food sources of dietary sodium in this sample of Victorian adults.

The large increase in sodium purchases from retail outlets between baseline and follow-up may be a consequence of changes in purchasing patterns consequent to Covid-19 lockdowns in Victoria. However, additional analyses of the data collected at follow-up before and after the lockdowns showed no difference in findings (Supplementary Table 4, sodium sourced from retail was 74% pre-lockdown vs 80% post-lockdown, *p* = 0.296). There are some international data suggesting Covid-19 did positively influence planning, selection and preparation of healthier foods related to perceived time availability and stay-at-home policies [45]. However, the current study was not designed to test this hypothesis, and the data collected were small and do not support those findings. The changes could also possibly be due to between-person variability of food consumed and within person daily variability of food consumed, compounded by weekend/weekday of dietary recall collection.

### Why was not there a significant impact in this sample population of Victorian adults?

Interventions to reduce sodium consumption at a population level necessitate complex interventions and a high degree of sustained action across multiple sectors impacting the food system. In the Australian context, food policy reflects the different responsibilities of federal and state governments and a wide variety of other agencies, interest groups and businesses.

An in-depth process evaluation is currently underway and will contribute further to understanding the effects of this initiative. A separate evaluation on consumer awareness campaign on adults’ self-reported knowledge, attitudes and behaviours revealed the campaign to have minimal impact in parents (target market) and the wider adult population; noting both groups already had a good knowledge of high salt intakes and the link to common health conditions; and an overall low reach of the primarily digitally delivered campaign [46]. An analysis of salt intake in Victorian primary school children also showed no change in salt intake after the 4-year state-wide population salt reduction intervention [20]. Notably, there were no salt reduction initiatives being led or coordinated nationally at the time of the intervention, and there had never previously been a state-level intervention of this type in Australia.

It could be hypothesised that the strategies in this initiative were not strong enough or perceived as relevant enough to shift consumer behaviour and had limited reach and the dose (intensity/length) was not enough on top of existing background health promotion campaigns to cut through to Victorian adults [21]. Progress with industry and voluntary reformulation is also a slow process and 4 years was possibly not long enough to see significant change in sodium content of processed foods. Despite strengths such as the diversity of stakeholders in the Victorian Salt Reduction Partnership and expertise in implementation, there were significant challenges to implementation perceived by 14 partnership stakeholders interviewed such as limited mediums of intervention delivery, inability to cut-through other nutrition messages in a crowded nutrition media space, misalignment of organisational and partnership views, unclear roles, responsibilities and authority, and salt not being a priority at state/federal levels [21]. Stakeholders did not think the political and social climate was conducive to prioritising salt as a population health issue and this was a barrier to achieving the Victorian Salt Reduction Partnership aims [21]. The state-based approach was also hindered by a lack of policy levers that the state government had access to compared to the Australian federal government [21]. State-wide and community salt reduction programs may be effective and often the decentralised (federal) decision-making process at the state level can allow for more rapid progress [47].

Decentralised decision-making may be an advantage at the community level, but for significant impact in food available at a population level in the state of Victoria, federal decision making (i.e. setting salt reduction targets for processed foods) is required. Scaling up state-wide efforts in conjunction with national initiatives is suggested to be the most effective and sustainable approach to reducing population salt intake[47] as there comes a point where a state lacks jurisdiction/power (e.g. reformulation regulations) to influence salt consumption due to federal laws and policies.

The current study’s findings add to evidence that national/federal government support is required for impactful and sustainable change. The implementation of the Victorian salt reduction initiative occurred when there were no national salt reduction initiatives occurring nationally—the Federal government’s Healthy Food Partnership sodium content targets were not released until after the intervention period—and despite a national nutrition labelling system (Health Star Rating (HSR)) being introduced in 2014 [48], it was not salt specific and analysis of products against HSR and Australian Dietary Guidelines in 2018 recommended a review of weighting given to salt in the HSR algorithm as many foods had a high HSR despite being high in salt [49].

The modest budget to deliver this initiative may also not have been enough—crude calculations of the budget against the Victorian population (~ $750,000 AUD/year over 5 years) equates to ~ 11c per person/year. The UK national salt reduction programme had a budget of £96 million over 10 years [50] and successfully reduced salt intake from 9.5 to 8.1 g/d [51]. Note, other than duration and size in budget, the UK salt reduction programme also had significant differences in initiative design, including top down national support with the Food Standards Agency setting salt reduction targets for a comprehensive range of food products (> 80 food category targets) [52]. Perhaps for Victoria, had there been support in setting salt reduction targets for manufacturers similar to UK, the 4-year period may have been long enough to see a change in salt consumption.

### Implications for future salt reduction initiatives

Urgent and accelerated action is required to achieve the targeted 30% reduction in salt intake within the Australian population [53]. There is an increasing quantity and quality of evidence-based strategies to guide salt reduction initiatives. An updated international review revealed the main implementation strategies were interventions in settings (e.g. schools, workplaces, fast food, restaurants, hospitals), food reformulation, consumer education, front of pack labelling and salt taxation [53]. Whilst there has been a reduction in consumer education approaches (which have modest effect), it is promising to see an increase in structural and policy-based initiatives (targets for salt levels in foods, food procurement policies or nutrition standards, front of pack labelling and salt taxation) are being implemented to create environments conducive to healthier food options and consequently reduce population salt intake [53].

Interventions aiming to reduce salt consumption are projected to be not only cost effective, but cost saving by substantially reducing cardiovascular disease burden and expenditure on health care [12]. Voluntary options at a population level to help improve the healthiness of diets (i.e. lower salt) have been trialled in Australia with little impact over time. For example, voluntary display of the HSR label which includes information on sodium labelling is provided on packaged food products; however, only 40% of eligible products (with a healthy-bias) are labelled [54]. Voluntary nutrient reformulation targets (sodium, saturated fat, sugar) on processed foods by the Federal Government’s Healthy Food Partnership also appear too conservative and may have limited effect on population health [22, 55, 56]. There is an urgent need for government to step up and implement mandatory and more stringent reformulation targets for sodium content permitted in processed foods in order to achieve greater public health benefits [55, 56].

Sodium reformulation targets, as part of a comprehensive national systems approach to address this complex problem is recommended. Ideally, a mix of top-down (policies, regulations, programs) and bottom-up (developed or tailored by the community to their local needs and contexts) is required [57]. A recent review revealed that multi-component strategies involving both upstream and downstream interventions, similar to those UK [9] have achieved the biggest reduction in population salt consumption [58]. Upstream population-wide policies include regulation, mandatory reformulation (which is much more effective than voluntary reformulation) and food labelling [58]. Changing individual behaviours and food choices is difficult, so changing the environment where individuals make their food choices can improve dietary intake and health [59]. Reformulation success, however, is dependent on the variety and extent to which products are reformulated [59]. A recent modelling study using grocery purchase data from nationally representative Australian households has suggested that national sodium reformulation targets (established post-the current salt reduction intervention) would only modestly reduce sodium per capita [− 50 mg/d (− 3.5%)], and this is achieved only if it is assumed 100% of manufacturers comply [60]. This is a very small reduction in sodium purchases and advocates for more stringent sodium reformulation targets [60]. Modelling further supports mandatory moderate salt limits for manufacturers; with 20 times the health benefits for the Australian population predicted compared to voluntary approaches [13].

Strategies to focus on in an Australian context could include nutrition labelling, mandatory reformulation targets and incentives, the use of salt substitutes (in reformulation and for discretionary salt behaviours), creating health promoting retail and food service environments and promotion of fresh, affordable, minimally process foods over highly processed foods which would not only reduce salt consumption, but improve overall diets. Furthermore, as Australia is a multi-cultural population, future studies should examine salt consumption, key food sources, origin and discretionary salt behaviours in an ethnically diverse sample to allow strategies to be culturally and contextually tailored. Comprehensive monitoring and evaluation of implemented strategies is also required, so lessons can be shared [53] and resources and efforts are used efficiently and not on reinventing the wheel. Investment now will outweigh health costs in future to overconsumption of salt. Future evaluation needs to consider timing of policy implementation, environmental impact and cultural changes.

## Strengths and limitations

The study has a number of strengths including the use of gold standard 24-h urinary sodium excretion to objectively measure the primary outcome of change in salt intake. Complementing this to provide information on food sources of salt was the use of a five-pass 24-h dietary recall conducted by a trained researcher in a subsample of participants. However, we do acknowledge several limitations. Foremost, despite best efforts at both time points, recruitment of participants was difficult, resulting in a smaller than expected sample size. The response rate was 7.7–8.5%, and which, whilst low, is similar to previous studies collecting 24-h urine excretions [61]. The study may have a healthy volunteer bias as participation was voluntary. The discretionary salt behaviour survey and anthropometric measurements were self-reported and may be subject to social desirability and self-report bias, respectively. Despite these limitations, these findings are useful to guide the next iteration of salt reduction strategies in Australia.

## Conclusion

In this Victorian population of adults, a 4-year state-wide salt reduction initiative had no impact on salt intake; sources of dietary sodium consumed or discretionary salt use. These data suggest a more intensive effort, supported at a national level and aimed at retail and food industry, is necessary given the large amount of sodium obtained from processed/ultraprocessed foods and retail settings.

## Supplementary Information

Below is the link to the electronic supplementary material.

Supplementary file1 (DOCX 30 KB)

Supplementary file2 (DOCX 14 KB)

Supplementary file3 (DOCX 13 KB)

Supplementary file4 (DOCX 18 KB)

Supplementary file5 (DOCX 14 KB)

## Data Availability

The mean data generated in the current study are included in this published article. The raw data used in this study cannot be made publicly available as participants did not provide consent for their data to be used for purposes other than described in the original study aims. The data that support the findings may be made available by the corresponding author for reasonable request upon approval by the Deakin University Human Research Ethics Committee.

## Abbreviations

BMI: Body Mass Index
BMR: Body Mass ratio
SDT: Suggested Dietary Target
SEIFA: Socio-Economic Indexes for Areas
UK: United Kingdom
WHO: World Health Organization

## References

[1] He FJ, Tan M, Ma Y, MacGregor GA (2020). Salt reduction to prevent hypertension and cardiovascular disease: JACC state-of-the-art review. J Am Coll Cardiol.

[2] National Health Survey: first results 2017–18, data customised using TableBuilder. https://www.abs.gov.au/statistics/health/health-conditions-and-risks/national-health-survey-first-results/latest-release. Accessed 24 July 2023

[3] Key statistics: cardiovascular disease. https://www.heartfoundation.org.au/bundles/for-professionals/key-stats-cardiovascular-disease. Accessed 24 July 2023

[4] Causes of death, Australia, catalogue number 3303.0. https://www.abs.gov.au/statistics/health/causes-death/causes-death-australia/2019. Accessed 24 July 2023

[5] World Health Organization Guideline (2012) Sodium intake for adults and children. Geneva 23658998

[6] Land MA, Neal BC, Johnson C, Nowson CA, Margerison C, Petersen KS (2018). Salt consumption by Australian adults: a systematic review and meta-analysis. Med J Aust.

[7] Beaglehole R, Bonita R, Horton R, Adams C, Alleyne G, Asaria P (2011). Priority actions for the non-communicable disease crisis. Lancet.

[8] Kontis V, Mathers CD, Rehm J, Stevens GA, Shield KD, Bonita R (2014). Contribution of six risk factors to achieving the 25×25 non-communicable disease mortality reduction target: a modelling study. Lancet.

[9] He FJ, Pombo-Rodrigues S, Macgregor GA (2014). Salt reduction in England from 2003 to 2011: its relationship to blood pressure, stroke and ischaemic heart disease mortality. BMJ Open.

[10] Laatikainen T, Pietinen P, Valsta L, Sundvall J, Reinivuo H, Tuomilehto J (2006). Sodium in the Finnish diet: 20-year trends in urinary sodium excretion among the adult population. Eur J Clin Nutr.

[11] Canada H (2018). Sodium intake of Canadians in 2017.

[12] Hope SF, Webster J, Trieu K, Pillay A, Ieremia M, Bell C (2017). A systematic review of economic evaluations of population-based sodium reduction interventions. PLoS ONE.

[13] Cobiac LJ, Vos T, Veerman JL (2010). Cost-effectiveness of interventions to reduce dietary salt intake. Heart.

[14] Jones A, Magnusson R, Swinburn B, Webster J, Wood A, Sacks G (2016). Designing a healthy food partnership: lessons from the Australian Food and Health Dialogue. BMC Public Health.

[15] Australian Bureau of Statistics (n.d.) Region summary:Victoria. https://dbr.abs.gov.au/region.html?lyr=ste&rgn=2. Accessed 24 July 2023

[16] VicHealth (2015) State of Salt: The case for salt reduction in Victoria. https://www.vichealth.vic.gov.au/media-and-resources/publications/state-of-salt. Accessed 24 July 2023

[17] Trieu K, Jan S, Woodward M, Grimes C, Bolam B, Nowson C (2018). Protocol for the process evaluation of a complex, statewide intervention to reduce salt intake in Victoria, Australia. Nutrients.

[18] VicHealth (2021) Salt reduction in Victoria. https://www.vichealth.vic.gov.au/programs-and-projects/salt-reduction. Accessed 24 July 2023

[19] The Heart Foundation (2021) How we unpacked the salt in Victoria. In. Melbourne, Victoria, Australia

[20] Grimes C, Bolton KA, Trieu K, Reimers J, Armstrong S, Bolam B, Beckford K, Santos JA, Rosewarne E, Dunford E (2023). Evaluation of a state-wide intervention on salt intake in primary schoolchildren living in Victoria, Australia. Public Health Nutr.

[21] Rosewarne E, Chislett WK, McKenzie B, Reimers J, Jolly KA, Corben K (2021). Stakeholder perspectives on the effectiveness of the Victorian Salt Reduction Partnership: a qualitative study. BMC Nutr.

[22] Rosewarne E, Moore M, Chislett WK, Jones A, Trieu K, Webster J (2021). An evaluation of the Victorian Salt Reduction Partnership's advocacy strategy for policy change. Health Res Policy Syst.

[23] Rosewarne E, Trieu K, Farrand C, Reimers J, Potter J, Davidson C (2020). Unpack the Salt: an evaluation of the Victorian Salt Reduction Partnership's media advocacy activities to highlight the salt content of different foods. Nutr J.

[24] Bolton KA, Webster J, Dunford EK, Jan S, Woodward M, Bolam B (2020). Sources of dietary sodium and implications for a statewide salt reduction initiative in Victoria, Australia. Br J Nutr.

[25] Land MA, Webster J, Christoforou A, Praveen D, Jeffery P, Chalmers J (2014). Salt intake assessed by 24 h urinary sodium excretion in a random and opportunistic sample in Australia. BMJ Open.

[26] Green P, Macleod CJ (2016). simr: an R package for power analysis of generalised linear mixed models by simulation. Methods Ecol Evol.

[27] Australian Bureau of Statistics (2018) 2016 Census QuickStats. https://quickstats.censusdata.abs.gov.au/census_services/getproduct/census/2016/quickstat/2?opendocument. Accessed 24 July 2023

[28] Nowson C, Lim K, Grimes C, O'Halloran S, Land MA, Webster J (2015). Dietary salt intake and discretionary salt use in two general population samples in Australia: 2011 and 2014. Nutrients.

[29] Australian Bureau of Statistics (2013) 4363.0.55.001 - Australian Health Survey: Users' Guide, 2011–13. https://www.abs.gov.au/AUSSTATS/abs@.nsf/DetailsPage/4363.0.55.0012011-13?OpenDocument. Accessed 24 July 2023

[30] Australian Bureau of Statistics (2010) Australian Health Survey food model booklet. Belconnen, ACT

[31] Centres for Disease Control and Prevention (2017) National Health and Nutrition Examination Survey. MEC In-Person Dietary Interviewers Procedures Manual

[32] Food Standards Australia New Zealand (2020) AUSNUT 2011–2013. http://www.foodstandards.gov.au/science/monitoringnutrients/ausnut/Pages/default.aspx. Accessed 24 July 2023

[33] World Health Organization (2010) Body mass index—BMI. http://www.euro.who.int/en/health-topics/disease-prevention/nutrition/a-healthy-lifestyle/body-mass-index-bmi. Accessed 24 July 2023

[34] Australian Bureau of Statistics (2022) Socio-Economic Indexes for Areas. http://www.abs.gov.au/websitedbs/censushome.nsf/home/seifa. Accessed 24 July 2023

[35] Forni Ogna V, Ogna A, Vuistiner P, Pruijm M, Ponte B, Ackermann D (2015). New anthropometry-based age- and sex-specific reference values for urinary 24-hour creatinine excretion based on the adult Swiss population. BMC Med.

[36] Cogswell ME, Loria CM, Terry AL, Zhao L, Wang CY, Chen TC (2018). Estimated 24-hour urinary sodium and potassium excretion in US adults. JAMA.

[37] Australian Government National Health and Medical Research Council (2016) Nutrient Reference Values for Australia and New Zealand—Sodium. https://www.nrv.gov.au/nutrients/sodium. Commonwealth of Australia. Accessed 24 July 2023

[38] Schofield WN (1985). Predicting basal metabolic rate, new standards and review of previous work. Hum Nutr Clin Nutr.

[39] Gibson R (2005). Principles of nutritional assessment Oxford.

[40] Moubarac JC, Parra DC, Cannon G, Monteiro CA (2014). Food classification systems based on food processing: significance and implications for policies and actions: a systematic literature review and assessment. Curr Obes Rep.

[41] O'Halloran SA, Lacy KE, Woods J, Grimes CA, Campbell KJ, Nowson CA (2018). The provision of ultra-processed foods and their contribution to sodium availability in Australian long day care centres. Public Health Nutr.

[42] National Health and Medical Research Council (2013). Australian guide to healthy eating.

[43] Australian Bureau of Statistics (2013) 4363.0.55.001—Australian Health Survey: Users' guide, 2011–13 Australian Health Survey—discretionary food list

[44] Krebs-Smith SM, Kott PS, Guenther PM (1989). Mean proportion and population proportion: two answers to the same question?. J Am Diet Assoc.

[45] De Backer C, Teunissen L, Cuykx I, Decorte P, Pabian S, Gerritsen S (2020). An evaluation of the COVID-19 pandemic and perceived social distancing policies in relation to planning, selecting, and preparing healthy meals: an observational study in 38 countries worldwide. Front Nutr.

[46] Grimes CA, Bolton KA, Lim K, Khokhar D, Santos JA, Trieu K, Margerison C, Reimers J, Armstrong S, Bolam B (2023). Evaluation of a salt-reduction consumer awareness campaign targeted at parents residing in the State of Victoria, Australia. Nutrients.

[47] Christoforou A, Trieu K, Land MA, Bolam B, Webster J (2016). State-level and community-level salt reduction initiatives: a systematic review of global programmes and their impact. J Epidemiol Community Health.

[48] Department of Health (2022) Health Star Rating System: Governance. http://healthstarrating.gov.au/internet/healthstarrating/publishing.nsf/Content/governance. Commonwealth of Australia. Accessed 24 July 2023

[49] Jones A, Radholm K, Neal B (2018). Defining 'unhealthy': a systematic analysis of alignment between the Australian Dietary Guidelines and the health star rating system. Nutrients.

[50] Collins M, Mason H, O'Flaherty M, Guzman-Castillo M, Critchley J, Capewell S (2014). An economic evaluation of salt reduction policies to reduce coronary heart disease in England: a policy modeling study. Value Health.

[51] Wyness LA, Butriss JL, Stanner SA (2012). Reducing the population's sodium intake: the UK Food Standards Agency's salt reduction programme. Public Health Nutr.

[52] Public Health England Salt Reduction Programme (2014) https://www.gov.uk/government/publications/salt-reduction-targets-for-2024. Crown copyright. Accessed 24 July 2023

[53] Santos JA, Tekle D, Rosewarne E, Flexner N, Cobb L, Al-Jawaldeh A (2021). A systematic review of salt reduction initiatives around the world: a midterm evaluation of progress towards the 2025 global non-communicable diseases salt reduction target. Adv Nutr.

[54] Shahid M, Neal B, Jones A (2020). Uptake of Australia's health star rating system 2014–2019. Nutrients.

[55] Rosewarne E, Huang L, Farrand C, Coyle D, Pettigrew S, Jones A (2020). Assessing the healthy food partnership's proposed nutrient reformulation targets for foods and beverages in Australia. Nutrients.

[56] Trieu K, Coyle DH, Afshin A, Neal B, Marklund M, Wu JHY (2021). The estimated health impact of sodium reduction through food reformulation in Australia: a modeling study. PLoS Med.

[57] Bolton KA, Kremer P, Gibbs L, Waters E, Swinburn B, de Silva A (2017). The outcomes of health-promoting communities: being active eating well initiative-a community-based obesity prevention intervention in Victoria, Australia. Int J Obes (Lond).

[58] Hyseni L, Elliot-Green A, Lloyd-Williams F, Kypridemos C, O'Flaherty M, McGill R (2017). Systematic review of dietary salt reduction policies: evidence for an effectiveness hierarchy?. PLoS ONE.

[59] Gressier M, Swinburn B, Frost G, Segal AB, Sassi F (2021). What is the impact of food reformulation on individuals' behaviour, nutrient intakes and health status? A systematic review of empirical evidence. Obes Rev.

[60] Coyle D, Shahid M, Dunford E, Ni Mhurchu C, McKee S, Santos M (2021). Estimating the potential impact of Australia's reformulation programme on households' sodium purchases. BMJ Nutr Prev Health.

[61] Land MA, Wu JH, Selwyn A, Crino M, Woodward M, Chalmers J (2016). Effects of a community-based salt reduction program in a regional Australian population. BMC Public Health.

[62] Australian Bureau of Statistics (2017) 3235.0 Regional Population by Age and Sex, Australia 2016. https://www.abs.gov.au/AUSSTATS/abs@.nsf/Lookup/3235.0Main+Features12016?OpenDocument. Commonwealth of Australia. Accessed 24 July 2023

[63] Department of Health and Human Services (2018) Victorian Population Health Survey 2016: selected survey findings. Melbourne

