# Supplementary material for: Outcomes of a state-wide salt reduction initiative in adults living in Victoria, Australia
Source: Eur J Nutr. 2023 Jul 26;62(7):3055–67. doi: 10.1007/s00394-023-03210-z (PMC10468945; doi:10.1007/s00394-023-03210-z)
Supplement: Supplementary file 1 — Supplementary file1 (DOCX 30 KB) [file 394_2023_3210_MOESM1_ESM.docx]

**Supplementary Figure 1 – flow chart of participants at baseline and follow-up**

**Baseline**

**Follow up**
