# Supplementary material for: Outcomes of a state-wide salt reduction initiative in adults living in Victoria, Australia
Source: Eur J Nutr. 2023 Jul 26;62(7):3055–67. doi: 10.1007/s00394-023-03210-z (PMC10468945; doi:10.1007/s00394-023-03210-z)
Supplement: Supplementary file 2 — Supplementary file2 (DOCX 14 KB) [file 394_2023_3210_MOESM2_ESM.docx]

**Supplementary Table 1: Response rates across the three recruitment strategies – baseline vs follow-up**

|  | Previous participants | | | Electoral participants | | O week participants | | Total | | |
| --- | --- | --- | --- | --- | --- | --- | --- | --- | --- | --- |
|  | 2016/7 | | 2021 | 2016/7 | 2021 | 2016/7 | 2021 | 2016/7 | | 2021 |
| Invited | 271 | | 375 | 5694 | 3000 | 204 | 164 | 6169 | | 3539 |
| Consented | 185 | | 153 | 175 | 100 | 102 | 47 | 462 | | 300 |
| Withdrew | 6 | | 5 | 11 | 8 | 24 | 7 | 41 | | 20 |
| Consented but urine and/or survey or no longer contactable | 8 | | 19 | 13 | 19 | 30 | 19 | 41 | | 57 |
| Return to sender | 9 | | 0 | 5 | 88 | 5 | 0 | 19 | | 88 |
| Did not meet study inclusion criteria | 6 | | 0 | 11* | 0 | 0 | 0 | 6 | | 0 |
| Deceased | 1 | | 2 | 0 | 0 | 0 | 0 | 1 | | 0 |
| Not required as age group quota full | 0 | | 55 | 4 | 0 | 0 | 0 | 4 | | 55 |
| Urine sample lost | 3 | | 0 | 2 | 0 | 0 | 0 | 5 | | 0 |
| Response rate | 68.3 | 40.8 | | 3.1 | 3.3 | 50.0 | 28.7 | 7.5 | 8.5 | |

*withdrew and counted in above for this (i.e. consented but did not meet inclusion criteria so formally withdrew)
