# Supplementary material for: Outcomes of a state-wide salt reduction initiative in adults living in Victoria, Australia
Source: Eur J Nutr. 2023 Jul 26;62(7):3055–67. doi: 10.1007/s00394-023-03210-z (PMC10468945; doi:10.1007/s00394-023-03210-z)
Supplement: Supplementary file 3 — Supplementary file3 (DOCX 13 KB) [file 394_2023_3210_MOESM3_ESM.docx]

**Supplementary Table 2: Completion of valid 24-hr urines and 24-hr diet recalls in relation to Covid-19 restrictions**

|  | **Collected prior to lockdown #1** | **Collected during lockdown #1** | **Collected between lockdown #1 and #2 (e.g. state was ‘reopened’)** | **Collected during lockdown #2** | **Total** |
| --- | --- | --- | --- | --- | --- |
|  | 16 March state of emergency, 20 March stay at home, 29 March | 30 March -30 May | 1 June-29 June | 30 June onwards |  |
| **Urines** | 137 (65%) | 52 (25%) | 14 (7%) | 8 (3%) | 211 |
| **Diet recalls** | 51 (57%) | 31 (34%) | 2 (2%) | 6 (7%) | 90 |

*****Note lockdowns were staged by regions and complicated to consolidate across the state. These dates are conservative to represent any possible change in purchasing and consumption behaviours.
