# Supplementary material for: Outcomes of a state-wide salt reduction initiative in adults living in Victoria, Australia
Source: Eur J Nutr. 2023 Jul 26;62(7):3055–67. doi: 10.1007/s00394-023-03210-z (PMC10468945; doi:10.1007/s00394-023-03210-z)
Supplement: Supplementary file 4 — Supplementary file4 (DOCX 18 KB) [file 394_2023_3210_MOESM4_ESM.docx]

The food sources of sodium by major groups are presented in Supplementary Table 3. At follow-up, cereal-based products and dishes, non-alcoholic beverages and dairy and meat substitutes contributed significantly more daily sodium compared to baseline (p<0.03). Meat, poultry and game products and dishes, and cereal and cereal products contributed significantly less daily sodium at follow-up compared to baseline (p<0.03).

**Supplementary Table 3: Contribution to daily sodium intake by major food groups as reported by 24-hour dietary recall**

|  | **Baseline (n=143)** | | | | **Follow-up (n=90)** | | | |  |
| --- | --- | --- | --- | --- | --- | --- | --- | --- | --- |
| **Major food group name** | **Rank** | **% of daily intake** | **95% CI** | | **Rank** | **% of daily intake** | **95% CI** | | **p value*** |
| Cereals and cereal products | 1 | 18% | 15.5 | 20.8 | 2 | 13% | 10.4 | 16.1 | 0.015 |
| Cereal-based products and dishes | 2 | 18% | 14.4 | 21.3 | 1 | 24% | 18.2 | 30.5 | 0.022 |
| Meat, poultry and game products and dishes | 3 | 17% | 13.2 | 19.8 | 3 | 10% | 6.5 | 14.1 | 0.009 |
| Milk products and dishes | 4 | 9% | 7.0 | 10.1 | 6 | 7% | 4.6 | 10.2 | 0.491 |
| Savoury sauces and condiments | 5 | 8% | 4.9 | 10.2 | 4 | 10% | 6.2 | 12.8 | 0.623 |
| Vegetable products and dishes | 6 | 6% | 4.7 | 8.0 | 5 | 8% | 4.1 | 11.6 | 0.682 |
| Soup | 7 | 5% | 2.6 | 7.0 | 8 | 4% | 0.6 | 6.8 | 0.438 |
| Non-alcoholic beverages | 8 | 5% | 4.1 | 6.1 | 7 | 6% | 4.7 | 7.1 | 0.022 |
| Miscellaneous | 9 | 2% | 0.7 | 3.2 | 10 | 2% | 0.8 | 3.8 | 0.814 |
| Legume and pulse products and dishes | 10 | 2% | 0.3 | 2.8 | 12 | 1% | 0.3 | 2.6 | 0.731 |
| Snack foods | 11 | 2% | 0.7 | 2.8 | 9 | 3% | 1.1 | 4.0 | 0.090 |
| Egg products and dishes | 12 | 1% | 0.8 | 1.9 | 13 | 1% | 0.3 | 1.6 | 0.669 |
| Alcoholic beverages | 13 | 1% | 0.6 | 1.4 | 14 | 1% | 0.3 | 0.8 | 0.086 |
| Fats and oils | 14 | 1% | 0.6 | 1.3 | 15 | 1% | 0.3 | 1.3 | 0.996 |
| Seed and nut products and dishes | 15 | 1% | 0.2 | 1.0 | 16 | 1% | 0.3 | 2.0 | 0.123 |
| Confectionery and cereal/nut/fruit/seed bars | 16 | 1% | 0.3 | 0.8 | 17 | 1% | 0.3 | 1.0 | 0.631 |
| Dairy and meat substitutes | 17 | 0% | 0.1 | 0.6 | 11 | 2% | 0.9 | 3.4 | 0.006 |
| Special dietary foods | 18 | 0% | 0.0 | 0.7 | 18 | 1% | -0.5 | 2.2 | 0.614 |

Data is only included if it contributes to ≥1% of intake at a baseline

*data displayed was calculated mean ratio and utilised in a mixed regression analysis adjusting for age, gender, BMI, SEIFA, energy (kj/day) and weekend/weekday collection.
