# Supplementary material for: Outcomes of a state-wide salt reduction initiative in adults living in Victoria, Australia
Source: Eur J Nutr. 2023 Jul 26;62(7):3055–67. doi: 10.1007/s00394-023-03210-z (PMC10468945; doi:10.1007/s00394-023-03210-z)
Supplement: Supplementary file 5 — Supplementary file5 (DOCX 14 KB) [file 394_2023_3210_MOESM5_ESM.docx]

**Supplementary Table 4:** **Sensitivity analysis examining if there was an impact of Covid on origin of purchase of food ingredients consumed as reported by 24-hour dietary recall**

|  | **Baseline (n=143)** | | | **All follow-up (n=90)** | | | | **Subsample of follow-up data collected PRE Covid lockdown (n=50)** | | | | **Subsample of follow-up data collected POST Covid lockdown (n=40)** | | | | **PRE vs POST Covid** |
| --- | --- | --- | --- | --- | --- | --- | --- | --- | --- | --- | --- | --- | --- | --- | --- | --- |
| **Origin of purchase** | **% daily intake** | **95% CI** | | **% daily intake** | **95% CI** | | **p value**  **baseline vs follow-up** | **% daily intake** | **95% CI** | | **p value baseline vs follow-up** | **% daily intake** | **95% CI** | | **p value baseline vs follow-up** | **p value** |
| Store - grocery/supermarket | 57 | 50.9 | 62.7 | 77 | 70.6 | 84.2 | <0.001 | 74 | 66.4 | 82.1 | <0.001 | 80 | 69.2 | 91.6 | <0.001 | 0.296 |
| Quick service/ take-out/delivery | 9 | 4.3 | 13.0 | 12 | 5.9 | 17.0 | 0.473 | 12 | 6.3 | 17.4 | 0.170 | 11 | 1.7 | 20.6 | 0.251 | 0.273 |
| Full service restaurant | 9 | 4.7 | 12.3 | 5 | 1.7 | 8.5 | 0.257 | 6 | 0.8 | 10.6 | 0.358 | 5 | -0.2 | 9.3 | 0.331 | 0.898 |
| Water from tap | 1 | 1.4 | 2.0 | 2 | 1.6 | 2.7 | 0.026 | 2 | 1.3 | 2.5 | 0.374 | 2 | 1.5 | 3.3 | 0.170 | 0.786 |
| Fresh food Market | 13 | 8.7 | 16.2 | 2 | 0.4 | 3.4 | <0.001 | 4 | 0.5 | 6.4 | <0.001 | 0.4 | -0.4 | 1.1 | <0.001 | 0.212 |
| From someone else/gift | 3 | 1.3 | 4.6 | 1 | 0.1 | 1.2 | 0.026 | 1 | 0.2 | 2.4 | 0.261 | 0 | 0 | 0 | Unable to run test | Unable to run test |
| Bar or tavern | 1 | 0.0 | 1.2 | 1 | -0.1 | 1.7 | 0.117 | 1 | -0.4 | 2.6 | 0.201 | 0.5 | -0.5 | 1.6 | 0.312 | 0.908 |
| Grown or caught | 3 | 1.3 | 4.9 | 0 | -0.1 | 0.4 | 0.004 | 0 | -0.01 | 0.1 | 0.001 | 0.3 | -0.3 | 0.8 | 0.147 | 0.360 |

Note: data displayed was calculated using mean ratio method and utilised in a mixed regression analysis adjusting for age, gender, BMI, SEIFA weekend/weekday collection.
